# ZeitZeiger: supervised learning for high-dimensional data from an oscillatory system

**DOI:** 10.1093/nar/gkw030

**Published:** 2016-01-26

**Authors:** Jacob J. Hughey, Trevor Hastie, Atul J. Butte

**Affiliations:** 1 Institute for Computational Health Sciences, University of California, San Francisco, San Francisco, CA 94158, USA; 2 Department of Statistics, Stanford University, Stanford, CA 94305, USA

## Abstract

Numerous biological systems oscillate over time or space. Despite these oscillators’ importance, data from an oscillatory system is problematic for existing methods of regularized supervised learning. We present ZeitZeiger, a method to predict a periodic variable (e.g. time of day) from a high-dimensional observation. ZeitZeiger learns a sparse representation of the variation associated with the periodic variable in the training observations, then uses maximum-likelihood to make a prediction for a test observation. We applied ZeitZeiger to a comprehensive dataset of genome-wide gene expression from the mammalian circadian oscillator. Using the expression of 13 genes, ZeitZeiger predicted circadian time (internal time of day) in each of 12 mouse organs to within ∼1 h, resulting in a multi-organ predictor of circadian time. Compared to the state-of-the-art approach, ZeitZeiger was faster, more accurate and used fewer genes. We then validated the multi-organ predictor on 20 additional datasets comprising nearly 800 samples. Our results suggest that ZeitZeiger not only makes accurate predictions, but also gives insight into the behavior and structure of the oscillator from which the data originated. As our ability to collect high-dimensional data from various biological oscillators increases, ZeitZeiger should enhance efforts to convert these data to knowledge.

## INTRODUCTION

Numerous biological systems oscillate over time or space, from metabolic oscillations in yeast (1) to the estrous cycle in mammals. Increasingly, these oscillatory biological systems are being quantified using ‘omics’ technologies, resulting in a growing number of high-dimensional datasets with periodic signals (2,3).

Given a dataset, one fundamental task is supervised learning, in which an algorithm learns the relationship between an input observation (a set of features) and an output variable. When performing supervised learning on ‘omics’ data, which typically have many more features than observations, a technique called regularization is often used to reduce model complexity and prevent overfitting (4). Although many methods have been developed for regularized supervised learning of standard continuous variables, the output variable of an oscillatory system is periodic, with no concept of low or high (e.g. time of day). This fundamental difference between the two types of variables means that methods designed for one cannot necessarily be applied to the other (5).

Recently, several methods have been developed for analyzing periodic data from single cells, particularly related to the cell cycle (6–8). However, in addition to being specific to either single-cell RNA-seq data or images of fixed cells, these methods are unsupervised. Thus, although valuable, these methods do not address the general problem of regularized supervised learning for periodic variables.

One oscillator present in species from cyanobacteria to humans is the circadian clock, which allows organisms to align their behavior to the time of day (9). In eukaryotes, the circadian clock is thought to be driven primarily by transcription-translation feedback loops between several genes and proteins (10–12). Mammals have a master clock in an area of the brain called the suprachiasmatic nucleus and a peripheral clock in almost every organ (13).

The periodic variable of the circadian clock, i.e. the internal time of day, is referred to as circadian time. Identifying molecules whose abundance is associated with circadian time has been the subject of many omics-based studies (14,15). Using omics data to predict circadian time, however, has received less attention (16–18).

To enable regularized supervised learning on high-dimensional data from an oscillatory system, we developed a method called ZeitZeiger. In the field of circadian rhythms, the term for an environmental cue that entrains the clock is *zeitgeber*, a German word that means ‘time giver.’ *Zeiger* in German refers to the hand of a clock and comes from the word *zeigen* (to show or reveal), so ZeitZeiger means ‘time revealer.’ ZeitZeiger learns a sparse representation of the variation associated with the periodic variable in the training observations, then uses maximum-likelihood to predict the value of the periodic variable for a test observation.

To demonstrate ZeitZeiger's utility, we applied it to 21 datasets of circadian gene expression in mice, comprising over 1000 samples, in order to train and validate a multi-organ predictor of circadian time. Our results suggest that ZeitZeiger can make accurate predictions, identify major patterns and important features, and detect when the oscillator is perturbed. Consequently, we expect that ZeitZeiger will be useful for analyzing data from a wide range of oscillatory systems. ZeitZeiger is available as an R package (https://github.com/jakejh/zeitzeiger), and all code, data and results for this study are available and reproducible (http://dx.doi.org/10.5061/dryad.hn8gp).

## MATERIALS AND METHODS

### Description of ZeitZeiger

**Figure 1.**
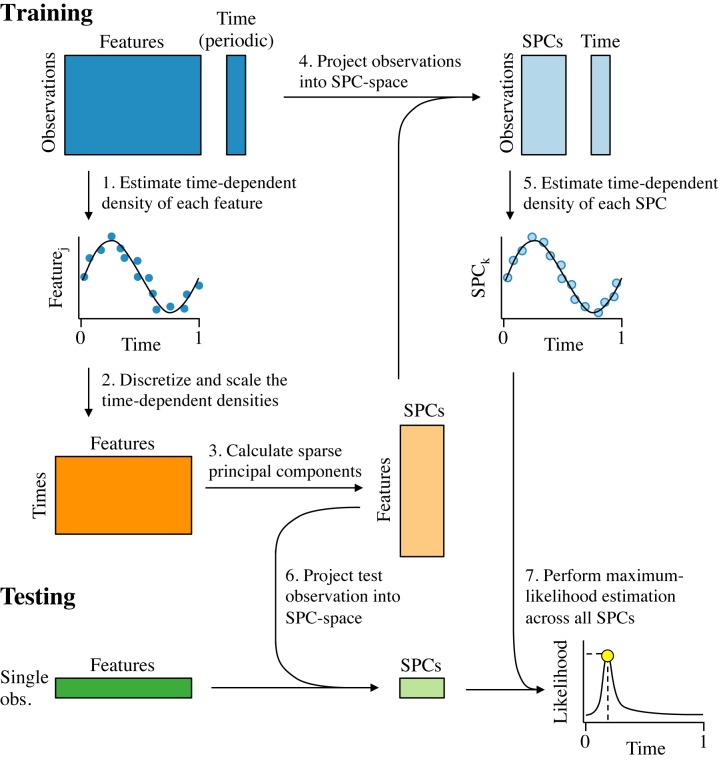
Schematic of the ZeitZeiger algorithm. The periodic variable is denoted as ‘time,’ with values between 0 and 1 and time = 0 equivalent to time = 1. Training data consist of a matrix of measurements for observations by features and a corresponding time for each observation. (**1**) The time-dependent mean of each feature is estimated as a smooth periodic spline and the variance about the mean is estimated based on the residuals. (**2**) A new matrix is constructed, in which the time-dependent mean of each feature is discretized into a number of time-points and scaled by that feature's standard deviation about the mean curve. (**3**) Sparse principal components (SPCs) of the new matrix are calculated. (**4**) The loadings of the features for each SPC are used to project the training data from feature-space into SPC-space. (**5**) The time-dependent mean and the variance of each SPC are estimated using the same procedure that was used for the features. (**6**) Each test observation is projected from feature-space into SPC-space. (**7**) Given the SPC values of the test observation and the time-dependent densities of the SPCs from the training data, the time of the test observation is predicted using maximum-likelihood.

ZeitZeiger (Figure 1) is a method to predict the value of a periodic variable, which we define as being continuous and bounded, where the maximum value is equivalent to the minimum value. For simplicity, we denote the periodic variable here as ‘time,’ but ZeitZeiger can be applied to any type of periodic measurement.

Similar to other supervised learning methods, training data should be a matrix $X \in \mathbb {R}^{n \times p}$ of measurements for *n* observations by *p* features and a vector $T \in \mathbb {R}^n$ of the corresponding time for each observation. ZeitZeiger assumes the density of each feature conditioned on time is Gaussian, so it is advisable to normalize the measurements accordingly. Time should be scaled between 0 and 1. Training data can have missing measurements. Test data cannot have missing measurements for the features used in the predictor (typically a small subset). Time-points in the training data do not have to be evenly spaced and each time-point could have a different number of replicates.

The first step of training is to estimate the time-dependent density of each feature *j* (step 1). Due to the nature of periodic variables, if a feature goes up, it must eventually come back down. To capture this non-monotonic behavior in an unbiased way, ZeitZeiger estimates the time-dependent mean, denoted *f*_*j*_(*t*), by fitting a periodic smoothing spline to the training observations (using the bigsplines R package (19)). Parameters of the spline, such as number of knots, can be adjusted as needed.

ZeitZeiger then estimates the variance of each feature, denoted $s^2_j$. Importantly, this is not simply the variance of the feature in the training observations, but the variance in the time-dependent density. By default, ZeitZeiger estimates the variance as the mean of the sum of squared residuals from the spline fit, i.e. $s^2_j = \frac{RSS_j}{n}$, so *s*_*j*_ is the estimated standard deviation about the mean curve. This assumes the variance of each feature about the mean is constant across time, which is simpler and more robust than trying to estimate a time-dependent variance (and seems to yield slightly more accurate predictions).

Next, ZeitZeiger identifies the major patterns that describe how the features change over time (steps 2 and 3). To do this, ZeitZeiger first constructs a matrix $Z \in \mathbb {R}^{m \times p}$ of time-points by features, in which the time-dependent mean of each feature is discretized into a number of time-points and scaled by that feature's standard deviation about the mean curve (step 2). The time-points are evenly spaced from 0 to 1, and the number of time-points *m* is adjustable. The value of *m* will be the maximum number of sparse principal components (SPCs) that can be used for prediction. If τ_*i*_ is the corresponding time-point for the *i*th row in *Z*, then

\begin{equation*} z_{ij} = \frac{f_j(\tau _i) - \bar{f}_j}{s_j}, \end{equation*}

where $\bar{f}_j$ is the mean of feature *j* over the selected time-points, calculated as:

\begin{equation*} \bar{f}_j = \frac{1}{m}\sum \limits _{i=1}^m f_j(\tau _i). \end{equation*}

Dividing by *s*_*j*_ ensures that each feature is expressed in terms of signal to noise.

ZeitZeiger then subjects *Z* to a penalized matrix decomposition (20) (PMD; step 3). By performing the PMD on *Z* and not on *X*, we are explicitly capturing the variation in the features associated with time (making ZeitZeiger conceptually similar to supervised principal components (21)). The right singular vectors from the PMD are the SPCs, which are linear combinations of a tunably small number of features. The SPCs are the source of ZeitZeiger's *L*_1_ regularization, the strength of which is controlled by the parameter *sumabsv*. By default, ZeitZeiger performs the PMD such that the left singular vectors are orthogonal to each other, which discourages the SPCs from being highly correlated with each other. We denote the matrix of *m* SPCs, each of length *p*, as $V \in \mathbb {R}^{p \times m}$. ZeitZeiger then uses the SPCs to project the training data from high-dimensional feature-space to low-dimensional SPC-space (step 4), producing a new matrix $\widetilde{X} \in \mathbb {R}^{n \times m}$ calculated as $\widetilde{X} = X V$.

In the last step of training, ZeitZeiger uses $\widetilde{X}$ to estimate the time-dependent density of each SPC in exactly the same way as was done for each individual feature (step 5). Although the time-dependent means of the SPCs could be extracted from the left singular vectors of the PMD, calculating the variances requires $\widetilde{X}$. We denote the time-dependent mean of the *k*th SPC as $\widetilde{f}_k(t)$ and the variance as $\widetilde{s}^2_k$.

Once the predictor is trained, making a prediction for a test observation $w \in \mathbb {R}^p$ requires only two steps. First, ZeitZeiger projects the test observation from feature-space to SPC-space: $\widetilde{w} = wV$ (step 6). Second, given the SPC values of the test observation and the estimated time-dependent densities of those SPCs from the training data, ZeitZeiger uses maximum-likelihood to predict the time of the test observation (step 7). Because we assume each SPC is Gaussian at any given time, the likelihood of time *t* given $\widetilde{w}_k$ is,

\begin{equation*} \ell _k(t \mid \widetilde{w}_k) = \frac{1}{\widetilde{s}_k\sqrt{2\pi }}e^{-(\widetilde{w}_k - \widetilde{f}_k(t))^2 / 2\widetilde{s}^2_k}. \end{equation*}

The final parameter of ZeitZeiger is *nSPC*, the number of SPCs used to calculate the likelihood, where *nSPC* ≤ *m*. Only features that contribute to at least one of the first *nSPC* SPCs will contribute to the prediction. If we treat the SPCs as if they were independent (which is not valid, but empirically works well), then the likelihood as a function of time is,

\begin{equation*} \ell (t \mid \widetilde{w}) = \prod \limits _{k=1}^{nSPC} \ell _k(t \mid \widetilde{w}_k) \end{equation*}

and the log-likelihood is,

\begin{equation*} L(t \mid \widetilde{w}) = \sum \limits _{k=1}^{nSPC} L_k(t \mid \widetilde{w}_k). \end{equation*}

The predicted time $\hat{t}$ for test observation *w* is,

\begin{equation*}_{\,\,\,\,\,\,\,\,\,\,\,\,\,_{t \in [0, 1)}}^{{\hat {t}}\,=\text{ arg max}\, L(t|{\widetilde {w}}).}\end{equation*}

To solve for $\hat{t}$, which is a bound-contrained optimization problem, ZeitZeiger uses the bbmle R package. For each test observation, ZeitZeiger provides the predicted time and the corresponding log-likelihood.

### Evaluating accuracy of predictions of a periodic variable

Calculating the prediction error of a periodic variable requires special care. We calculate the error $err(t, \hat{t})$ between an actual time *t* and a predicted time $\hat{t}$, where *t* ∈ [0, 1) and $\hat{t} \in [0, 1)$, as follows.

\begin{equation*} err(t, \hat{t}) = \left\lbrace \begin{array}{@{}l@{\quad }l@{}}\hat{t} - t, & \text{if } -0.5 \le \hat{t} - t \le 0.5 \\ \hat{t} - t + 1, & \text{if } \hat{t} - t < -0.5 \\ \hat{t} - t - 1, & \text{if } \hat{t} - t >0.5 \end{array}\right. \end{equation*}

This procedure makes the error as close to zero as possible. As a result, the error will always be between −0.5 and 0.5 and the absolute error will always be between 0 and 0.5. This implies that the absolute error of a random predictor follows a uniform distribution between 0 and 0.5, with a mean of 0.25 (6 h, if time is on a scale of 0 to 24 h).

Although we denote the difference between predicted circadian time and actual circadian time as ‘error,’ this assumes that external time (i.e. relative to the zeitgeber) is equal to the true circadian time (relative to the circadian clock). In light:dark cycles, this assumption should be approximately valid on average. However, in individual animals, the clock may not always exactly align with the external cue. Furthermore, in constant darkness, the free-running period of mice is slightly <24 h, so external time moves more slowly than true circadian time. Because we have no ground truth for circadian time in these datasets, we evaluate our predictions with respect to external time.

### Implementing the molecular-timetable method

The molecular-timetable was implemented based on Ueda *et al*. (16), with one change. Ueda *et al*. originally defined genes with high variability in expression using the coefficient of variation (standard deviation divided by the mean). However, our gene expression data contained negative values, making the coefficient of variation meaningless. Instead, we simply used the standard deviation.

### Processing microarray data for ZeitZeiger

All datasets (Supplementary Table S1) were processed as previously described (22) (https://github.com/jakejh/metapredict). Briefly, if raw Affymetrix data were available, expression values were normalized using RMA (23) and mapped to Entrez Gene IDs using customCDF (24). Otherwise, processed and normalized data were mapped to Entrez Gene IDs using the R package org.Mm.eg.db. Circadian time for each sample was standardized to be between 0 and 24 h, where CT0 marks ‘lights on’ or the beginning of subjective day.

### Applying ZeitZeiger to GSE54650

After processing the data for GSE54650 as described above, ComBat was used to adjust for organ-specific expression. ComBat is typically used to correct for batch effects between datasets, and from the perspective of the multi-organ predictor, differences in expression between organs are batch effects. Altogether, expression data from GSE54650 consisted of 21 115 genes measured in 288 samples. Other than to make the folds for cross-validation, ZeitZeiger was given no information about which samples came from which organ. As described in the main text, during cross-validation, we used a range of values for the two main parameters of ZeitZeiger, *sumabsv* and *nSPC*. Because gene expression in GSE54650 was measured every 2 h, we computed the SPCs using 12 time-points (*m* = 12).

### Applying the multi-organ predictor of circadian time to independent datasets

For each independent dataset, gene expression from GSE54650 and the independent dataset were merged as previously described (22). Briefly, expression data were reduced to the set of Entrez Gene IDs measured on both datasets, then ComBat was used to perform cross-study and cross-organ normalization. For datasets that contained genetic mutants, ComBat was also provided genotype as a covariate. Using ZeitZeiger, a predictor was trained on samples from GSE54650 and tested on samples from the independent dataset. As with cross-validation, the predictor was based solely on gene expression and had no information about which samples came from which organ.

## RESULTS

### Applying ZeitZeiger to a comprehensive dataset of circadian gene expression

To demonstrate ZeitZeiger's utility, we sought to use gene expression to predict the periodic variable of the circadian clock, referred to as circadian time (CT, where CT0 corresponds to sunrise). We applied ZeitZeiger to the most comprehensive dataset of circadian gene expression available (GSE54650; ref. (25)). GSE54650 contains 288 samples, consisting of 12 mouse organs sampled every 2 h for 2 days. For each sample, gene expression was measured for 21 115 genes (Affymetrix Mouse Gene 1.0 ST microarray). The mice were initially entrained to a 12:12 h light:dark cycle (LD 12:12), then released into constant darkness (DD) 18 h before the first samples were collected. By applying ZeitZeiger to GSE54650, we hoped to not only accurately predict circadian time, but also to identify a universal signature of the mouse circadian clock, one based on genes whose expression shows a consistent circadian rhythm in all tissues.

To determine the optimal parameter values for training the predictor, we first performed leave-one-organ-out cross-validation. For each organ, we trained a ZeitZeiger predictor on samples from the other 11 organs, and predicted the circadian time of each sample from the left-out organ. We used a range of values for each of the two main parameters of ZeitZeiger, *sumabsv* and *nSPC*. The first parameter, *sumabsv*, controls how many features (in this case, genes) form each SPC. The second parameter, *nSPC*, controls how many SPCs are used for prediction. Larger values of either parameter lead to a predictor based on more features. Prior to running cross-validation, we used ComBat (26) to adjust for organ-specific differences in gene expression, treating each organ as a batch (Supplementary Figure S1).

To evaluate the accuracy of a prediction, we used two metrics: error and absolute error. We calculated the error as the difference between predicted and observed CT, such that the error can range from −12 to +12 h (‘Materials and Methods’ section). Absolute error can then range from 0 to 12 h.

**Figure 2.**
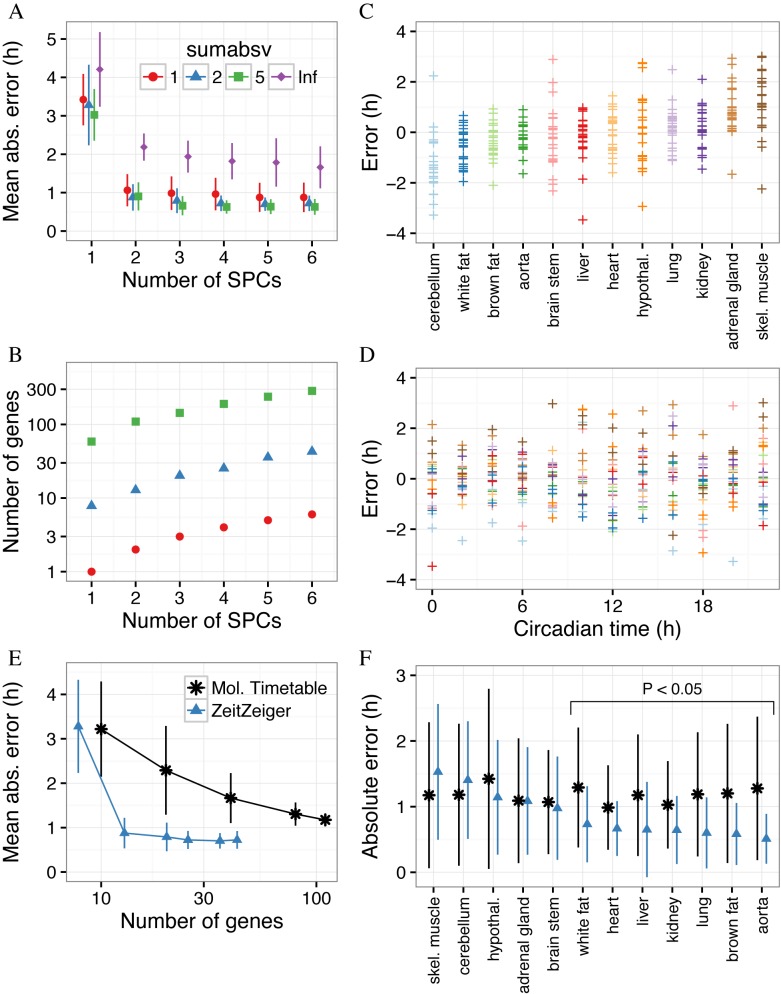
Using ZeitZeiger to predict circadian time of samples from GSE54650 in leave-one-organ-out cross-validation. (**A**) Overall mean absolute error (MAE) on cross-validation, as a function of the two main parameters of ZeitZeiger, *sumabsv* and *nSPC*. The point shows the overall MAE across all 288 samples and the error bar shows the standard deviation of the MAE across the 12 organs. *sumabsv* = *Inf* refers to a predictor trained using standard principal components instead of SPCs. (**B**) Mean number of genes in the predictors from cross-validation as a function of *sumabsv* and *nSPC*. (**C**) Prediction error (predicted CT minus observed CT) for each organ in GSE54650. Each point is a sample. Organs are sorted by mean error. (**D**) Prediction error as a function of circadian time. Each point is a sample, with color corresponding to organ, as in **C**. (**E**) Mean absolute error versus number of genes for ZeitZeiger and the molecular-timetable method. For ZeitZeiger, the predictor was trained using *sumabsv* = 2 and various values of *nSPC*. For the molecular-timetable method, various numbers of genes matching criteria for periodicity and variability were randomly selected. Each point shows the overall MAE across all 288 samples. The error bar shows the standard deviation of the MAE across the 12 organs. Points are connected by straight lines for ease of visualization. (**F**) Mean absolute error in each organ for ZeitZeiger and the molecular-timetable method. Organs are sorted by decreasing mean absolute error for ZeitZeiger. Statistical significance was evaluated using a two-sided, paired permutation test.

For each set of values of *sumabsv* and *nSPC* from leave-one-organ-out cross-validation, we calculated the mean absolute error (MAE) across all 12 organs (Figure 2A). The expected MAE of a completely random predictor is 6 h. Using only the first SPC (i.e. *nSPC* = 1), the MAEs of the ZeitZeiger-derived predictors with regularization were between 3 and 3.5 h. Using the first two SPCs (*nSPC* = 2) markedly improved accuracy, whereas including additional SPCs led to only small improvements. For predictors using at least the first two SPCs and any tested value of *sumabsv*, the MAEs on cross-validation were between 0.6 and 1.1 h. Importantly, training a predictor with regularization, i.e. using SPCs instead of standard principal components (denoted as *sumabsv* = *Inf* in Figure 2A), lowered the MAE by about 1 h. These results suggest that regularization in ZeitZeiger improves prediction accuracy of a periodic variable.

Instead of calculating the SPCs from a matrix derived from the training data, a simpler procedure would be to calculate the SPCs from the training data directly. To evaluate the performance of this simpler procedure, we again performed leave-one-organ-out cross-validation (Supplementary Figure S2). The simpler procedure led to predictions that were significantly less accurate than those by ZeitZeiger (*P* < 10^−5^ by two-sided, paired permutation test of the absolute error) for every tested value of *sumabsv* and *nSPC*, with an MAE anywhere from ∼1 to 5 h higher. These results suggest that ZeitZeiger's strategy of explicitly identifying the variation associated with the periodic variable, rather than all the variation in the training data, is superior for making predictions.

In addition to evaluating parameter sets in terms of prediction accuracy, we also evaluated them in terms of the number of genes used for prediction (Figure 2B). Remarkably, the predictor trained with *sumabsv* = 1 and *nSPC* = 2, whose MAE on cross-validation was about 1.1 h, was based on the expression of only two genes. Furthermore, the predictor trained with *sumabsv* = 2 and *nSPC* = 2, whose MAE was about 0.9 h, was based on the expression of only 13 genes on average.

We next examined whether the accuracy of ZeitZeiger's predictions varied by organ or by observed CT, focusing on the parameter values *sumabsv* = 2 and *nSPC* = 2 (Figure 2C and D). In cerebellum, predicted CT tended to be slightly behind observed CT (mean error −1.2 h, MAE 1.4 h), whereas in skeletal muscle, predicted CT tended to be slightly ahead of observed CT (mean error 1.3 h, MAE 1.5 h). All other organs had a mean error between −0.6 and 1 h, and an MAE <1.2 h (Supplementary Figure S3). In addition, predictions were similarly accurate across all observed CTs (Figure 2D; Supplementary Figure S4).

GSE54650 is an ideal dataset, because of its large sample size (288) and high time resolution (12 time-points per 24 h). To evaluate ZeitZeiger's performance in less-than-ideal scenarios, we split GSE54650 into training sets with various numbers of samples and time-points (Supplementary Figure S5). Given a training set of only 12 samples from either 3 or 4 time-points per 24 h, ZeitZeiger achieved a median absolute error (on test samples) of about 1 h. Taken together, our results suggest that even with relatively few training samples and low time resolution, ZeitZeiger can use the expression of a small number of genes to accurately predict circadian time in multiple mouse organs.

### Benchmarking ZeitZeiger against the molecular-timetable method

To benchmark ZeitZeiger against the state-of-the-art approach, we performed leave-one-organ-out cross-validation using the molecular-timetable method (MT; ref. (16)). MT was first developed to predict circadian time from gene expression in mouse liver, and has since been used to predict circadian time based on blood metabolite levels in mice and in humans (17,18). MT trains a predictor by selecting features that have high periodicity (i.e. high correlation with a cosine curve of period 24 h at any phase angle) and overall high variability. We used similar criteria for periodicity and variability to those used by Ueda *et al*. (16). Given a test observation, MT predicts circadian time by comparing the estimated time of peak expression of the selected genes with the expression of those genes in the test observation.

We compared MT and ZeitZeiger (*sumabsv* = 2, *nSPC* = 2) in terms of accuracy, number of selected genes and runtime. Predictions by MT had an overall MAE of 1.2 h, 34% higher than those by ZeitZeiger (*P* = 10^−5^ by two-sided, paired permutation test). ZeitZeiger's predictions were significantly more accurate than those by MT in 7 of 12 organs (unadjusted *P* < 0.05 by two-sided paired permutation test) and statistically indistinguishable in the others (Figure 2F). ZeitZeiger's predictions were ∼2–4× more accurate, when given a training set with few samples and low time resolution (Supplementary Figure S5). Impressively, ZeitZeiger achieved higher accuracy despite using markedly fewer genes (13 compared to 110 used by MT; Figure 2B and E).

To test whether MT could make accurate predictions with fewer genes, we followed the strategy of Ueda *et al*., randomly selecting subsets of genes that met the criteria for periodicity and variability (16). Consistent with their original observations, as the number of selected genes decreased, prediction accuracy also decreased (Figure 2E). When MT was restricted to only 10 genes, the MAE was ∼3.2 h.

Finally, ZeitZeiger was also more than twice as fast as MT. To run leave-one-organ-out cross-validation, MT required 69.8 min, whereas ZeitZeiger required only 30.4 min (both runtimes measured using a single core). In summary, compared to MT in predictions of circadian time based on gene expression, ZeitZeiger was faster and more accurate using fewer genes.

### Insights from the SPCs and genes that form the multi-organ predictor

Based on the results of cross-validation, we used the parameters *sumabsv* = 2 and *nSPC* = 2 to train a predictor on all samples from GSE54650. For the remainder of the paper, we call this the multi-organ predictor of circadian time. We then explored the properties of the multi-organ predictor in terms of SPCs and genes.

**Figure 3.**
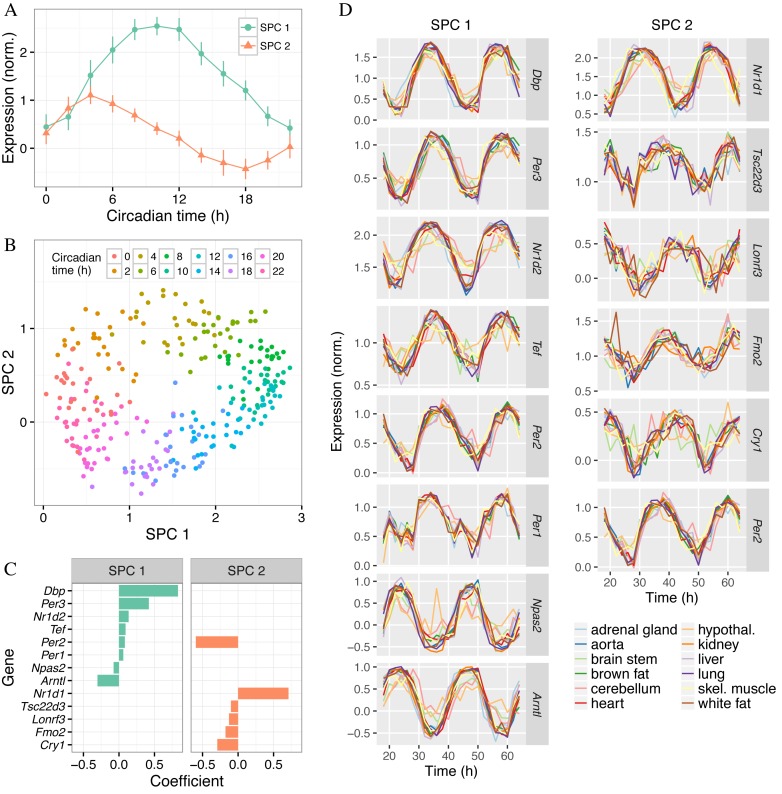
Properties of the SPCs and genes that form the multi-organ predictor. The predictor was trained on all samples of GSE54650 using *sumabsv* = 2 and *nSPC* = 2. (**A**) Expression of the two SPCs as a function of circadian time. The point shows the mean and the error bar shows the standard deviation across all samples at that circadian time. (**B**) Gene expression of the samples in SPC-space. Each point is a sample, with color indicating the circadian time. (**C**) Genes and their coefficients for the two SPCs. Only genes with non-zero coefficients are shown. Within each SPC, genes are sorted by their respective coefficients. (**D**) Expression versus time for the selected genes. Time is shown as the full 48 h of the experiment. Expression values shown are those obtained after adjusting for organ-specific expression using ComBat. Within each SPC, genes are sorted by their respective coefficients.

The SPCs are designed to explain the variation in the training data that is associated with the periodic variable. The two SPCs of the multi-organ predictor explained over 80% of the variance in circadian time-dependent gene expression in GSE54650, whereas no other SPC explained more than 5% (Supplementary Figure S6A). This is consistent with the fact that prediction accuracy in cross-validation did not substantially improve when *nSPC* increased past *nSPC* = 2 (Figure 2A). In addition, the ‘expression’ patterns of the SPCs (each of which is a linear combination of several genes) with respect to circadian time were sinusoidal and shifted from each other by about 6 h. When each sample from GSE54650 was plotted in SPC-space (SPC2 versus SPC1), the points described a cycle for which the progression of circadian time followed a clockwise trajectory (Figure 3B). These results imply that the circadian clock can be reasonably well approximated as a two-dimensional oscillator.

We next investigated the genes that formed the two SPCs (Figure 3C). Of the 13 genes, 8 are known to be part of the core circadian clock: *Arntl* (*Bmal1*), *Cry1, Per1, Per2, Per3, Nr1d1* (*Rev-erbα*), *Nr1d2* (*Rev-erbβ*) and *Npas2* (27). Two others, *Dbp* and *Tef*, are transcription factors regulated by the core clock that mediate circadian expression of many downstream genes (28). The three remaining genes (*Fmo2, Lonrf3, Tsc22d3*) have a less documented connection to the circadian clock. As we had hoped when applying ZeitZeiger to GSE54650, each gene showed a consistent circadian rhythm in each organ (Figure 3D). In addition, genes with larger coefficients for their respective SPCs tended to show stronger oscillations than genes with smaller coefficients, and genes with negative coefficients had inverted oscillations compared to genes with positive coefficients.

Finally, we compared the variation in circadian gene expression between organs with the variation in prediction of circadian time between organs. We observed that the circadian oscillations in brain stem, cerebellum and hypothalamus were relatively weak (Supplementary Figure S6B), which explains why the MAE for those organs was relatively high (Supplementary Figure S3). In addition, expression of some genes in cerebellum (particularly *Arntl* and *Nr1d2*) lagged behind expression in other organs, which explains cerebellum's negative mean error. In summary, the SPCs and genes selected by ZeitZeiger reveal both universal and organ-specific properties of the mouse circadian clock.

### The multi-organ predictor is accurate on multiple independent datasets

To validate ZeitZeiger and the multi-organ predictor, we performed a meta-analysis of circadian gene expression using nine additional datasets (Table 1; Supplementary Table S1; ref. (29–37)). These datasets differed in multiple ways from GSE54650: some measured expression in different organs or at different circadian times, and some used mice on a 12:12 h light:dark cycle (LD 12:12). Following the procedure we previously developed for meta-analysis of gene expression (22), each validation dataset was independently merged with GSE54650, then ZeitZeiger was used to train the predictor on samples from GSE54650 and predict circadian time of samples from the respective validation dataset. For GSE59396, we analyzed the DD and LD samples separately from each other.

**Table 1.** Description of datasets that include samples from wild-type mice

Dataset	Reference	Tissue	Samples	Light:dark regimen
E-MEXP-3780	Gossan *et al*. (2013)	metasternum	11	DD
GSE4238	Oster *et al*. (2006)	adrenal gland	24	DD
GSE10644	Hoogerwerf *et al*. (2008)	distal colon	18	LD 12:12
GSE11516	Na *et al*. (2009)	liver	36	LD 12:12
GSE11923	Hughes *et al*. (2009)	liver	48	DD
GSE25585	Keller *et al*. (2009)	macrophages	12	DD
GSE35795	Negoro *et al*. (2012)	bladder	12	DD
GSE38625	Geyfman *et al*. (2012)	skin	26	LD 12:12
GSE59396	Haspel *et al*. (2014)	lung	72	DD, LD 12:12

**Figure 4.**
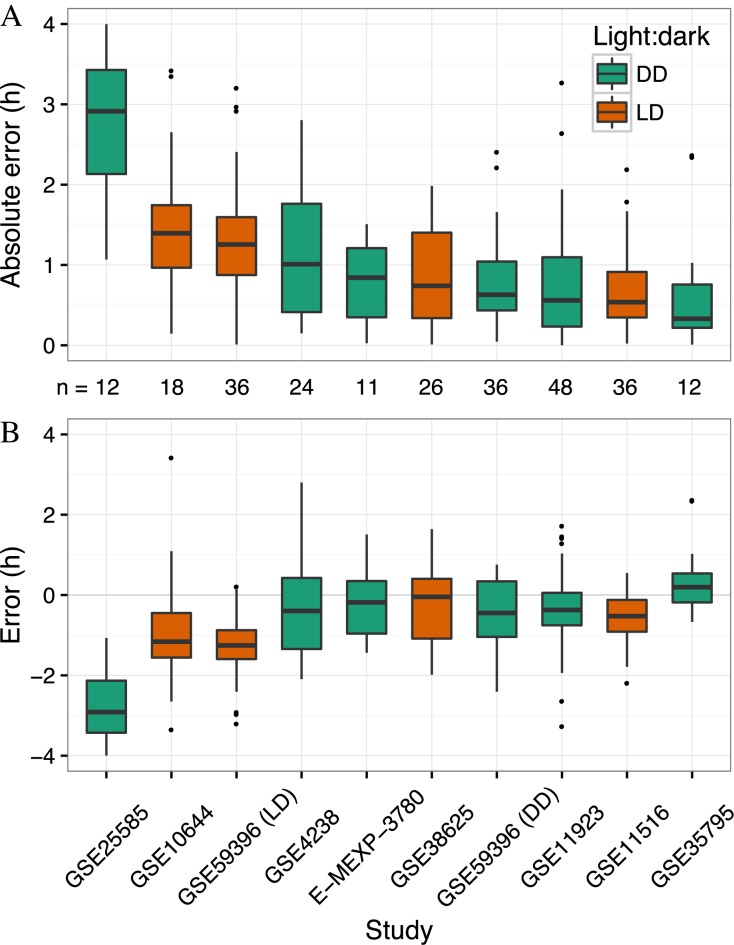
Applying the multi-organ predictor to independent datasets of circadian gene expression in wild-type mice. Boxplots of (**A**) absolute error and (**B**) error for each independent dataset. GSE59396 contains samples from DD and LD, so those are analyzed separately. Datasets are sorted by median absolute error. The number of samples in each dataset is indicated between panels **A** and **B**. For ease of visualization, one outlier has been omitted from each of GSE38625, GSE59396 (DD) and GSE59396 (LD). All points, including outliers, are visible in Supplementary Figure S7.

Despite the differences between GSE54650 and the other datasets, the median absolute error was <1.5 h in 9 of 10 datasets (including GSE59396 DD and LD; Figure 4A). Thus, the accuracy of the multi-organ predictor on independent datasets is similar to its accuracy on cross-validation of GSE54650. These results indicate that the multi-organ predictor can accurately and robustly predict circadian time using *in vivo* gene expression.

Interestingly, in nine of ten datasets, the median error was less than zero, which indicates a tendency for predicted CT to lag behind observed CT (Figure 4B; Supplementary Figure S7). Furthermore, in GSE59396, the error was significantly more negative for LD samples than for DD samples (*P* = 0.02 by two-sided *t*-test). This lag, which seems to be larger for LD compared to DD, may be due to the free-running period of C57BL/6J mice being slightly <24 h (38).

### The multi-organ predictor detects progression of the clock in cells cultured *in vitro*

To determine whether the multi-organ predictor could also be applied to cells cultured *in vitro*, we analyzed gene expression from mouse fibroblasts that were treated with molecules that synchronize the cells’ circadian clocks (Supplementary Table S1; ref. (33,39)). We hypothesized that in such synchronized cells, the multi-organ predictor would detect a linear progression of circadian time with time since synchronization. Indeed, for both NIH3T3s treated with forskolin (GSE11922) and mouse embryonic fibroblasts treated with dexamethasone (GSE49638), predicted CT increased approximately linearly with time since synchronization (Supplementary Figure S8). We conclude that the multi-organ predictor, trained on gene expression from mouse tissues *in vivo*, can detect progression of the circadian clock in cells cultured *in vitro*.

### The multi-organ predictor detects when the clock is phase-shifted

Our results thus far indicated that the multi-organ predictor is robust when the clock is operating normally. We next analyzed two datasets containing samples from mice in which the clock was phase-shifted (Supplementary Table S1; ref. (40,41)). We hypothesized that a phase shift would cause a systematic difference between predicted and observed CT, i.e. a systematically non-zero error.

In GSE13093, mice were fed either (i) ad libitum during entrainment then fasted in constant darkness or (ii) only between circadian times CT1 and CT9 in entrainment and in constant darkness (40). The latter condition is known to shift the phase of the circadian clock in peripheral tissues (42). Indeed, samples from fasted mice showed errors near zero, whereas samples from mice fed between CT1 and CT9 showed errors of ∼12 h (Supplementary Figure S9A and B).

In GSE52333, mice were fed either normal chow or a high fat diet, the latter of which caused the circadian rhythm of many transcripts and metabolites in the liver to undergo a phase advance (41). Accordingly, samples from mice fed a high fat diet showed errors ∼2 h higher than those of samples from mice fed normal chow (Supplementary Figure S9A and B). For both GSE13093 and GSE52333, the log-likelihoods of predicted CT were similarly high for both dietary conditions (Supplementary Figure S9C), suggesting that although the dietary perturbations shifted the phase of the clock, they did not impair its operation. These results indicate that the multi-organ predictor can accurately identify when the circadian clock is phase-shifted.

### The multi-organ predictor detects when the clock is genetically perturbed

To determine whether the multi-organ predictor could also recognize when the circadian clock is dysfunctional, we assembled a final group of seven datasets (Table 2; Supplementary Table S1; ref. (40,43–48)). Each dataset included samples from wild-type mice and from mice in which at least one component of the clock was knocked out or interfered with, either in the entire animal or in a specific tissue. Altogether, the seven datasets included five genetic mutations, expression from five organs and both DD and LD regimens.

**Table 2.** Description of datasets that include samples from mice with a genetically perturbed circadian clock

Dataset	Reference	Genetic mutation	Tissue of mutation	Tissue of gene expression	Samples	Light:dark regimen
GSE10045	Bray *et al*. (2008)	*MHCa:Clock*^Δ19^	cardiomyocyte	heart	130	LD 12:12
GSE13093	Vollmers *et al*. (2009)	*Cry1^-/-^ Cry2^-/-^*	whole organism	liver	64	DD
GSE27366	Nikolaeva *et al*. (2012)	*Clock^-/-^*	whole organism	kidney	22	DD
GSE34018	Cho *et al*. (2012)	*Nr1d1^-/-^ Nr1d2^-/-^*	liver	liver	24	LD 12:12
GSE35026	Paschos *et al*. (2012)	*Arntl^-/-^*	fat	fat	24	DD
GSE43071	Dyar *et al*. (2014)	*Arntl^-/-^*	skeletal muscle	calf	72	LD 12:12
GSE43073	Young *et al*. (2014)	*Arntl^-/-^*	cardiomyocyte	heart	64	LD 12:12

**Figure 5.**
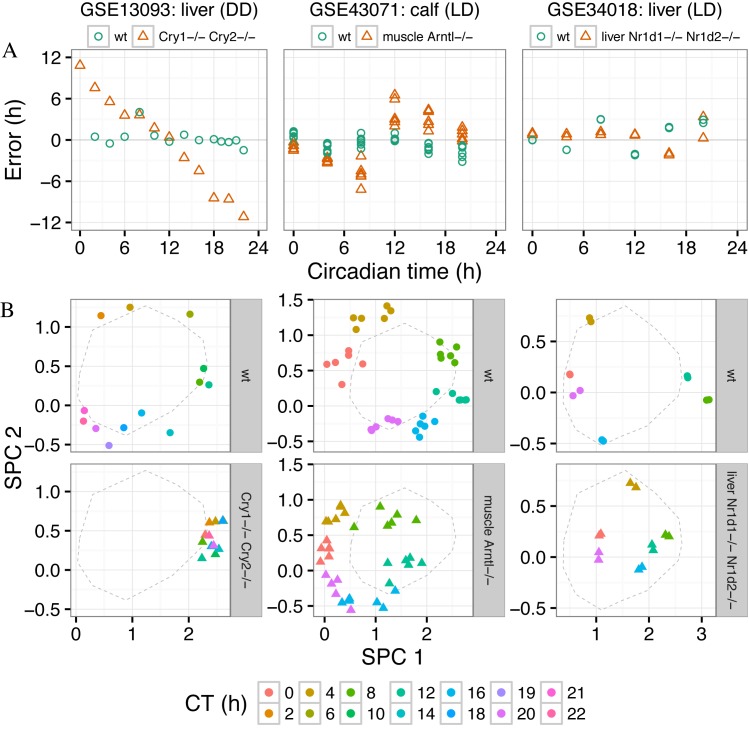
Applying the multi-organ predictor to gene expression from mice with a genetically perturbed circadian clock. The title of each column indicates the dataset, the organ in which gene expression was measured, and the light-dark regimen. The legend indicates the genetic mutation for that dataset, including whether the mutation is tissue-specific. In all plots, each point is a sample. Here we show results for only three datasets; Supplementary Figure S10 shows the results for all datasets. (**A**) Prediction error versus circadian time. (**B**) Gene expression of wild-type (upper) and mutant (lower) samples in SPC-space. The color of the point corresponds to the circadian time for that sample. The dashed line shows the mean trajectory of the training samples from GSE54650. The mean trajectory of the training samples is slightly different for each dataset, because each dataset was merged with GSE54650 separately, causing slight differences in the cross-study normalization.

**Figure 6.**
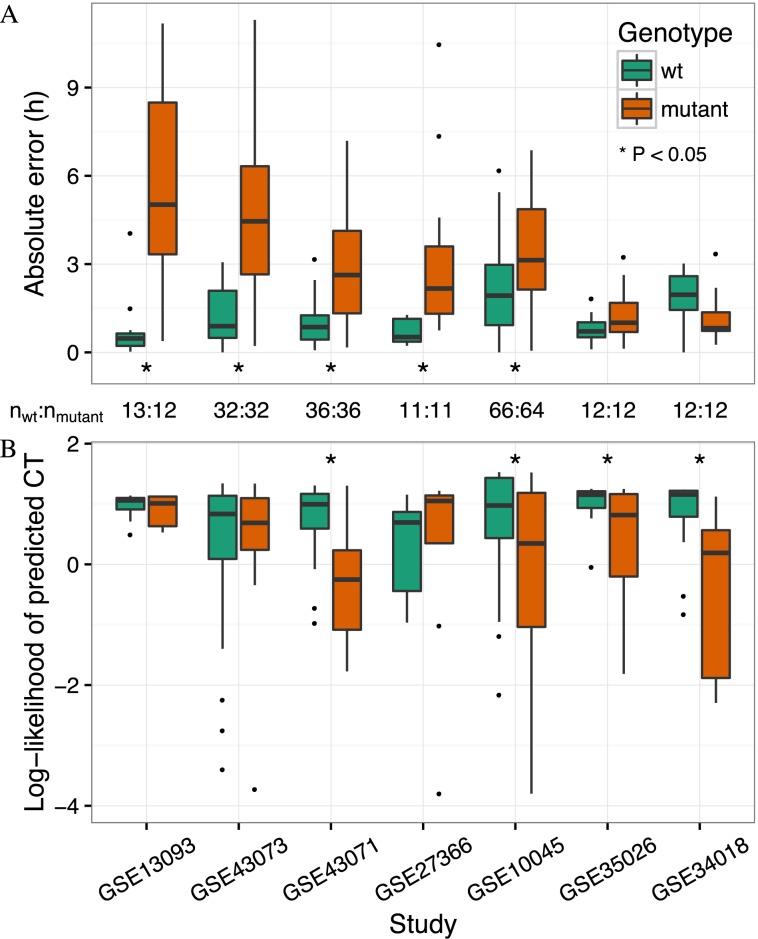
ZeitZeiger detects dysfunctional circadian gene expression caused by various genetic mutations. See Table 2 for a description of each dataset. Datasets are sorted by the difference in median absolute error between wild-type and mutant samples. The number of samples for each dataset is indicated between **A** and **B**. Statistical significance was evaluated using a one-sided t-test. (**A**) Boxplots of absolute error for wild-type and mutant samples. (**B**) Boxplots of log-likelihood of predicted circadian time for wild-type and mutant samples. For ease of visualization, a few outliers have been omitted from **B**. All samples, including outliers, are visible in Supplementary Figure S10.

We first compared mutant and wild-type samples in each dataset in terms of timing, i.e. predicted CT and error as a function of observed CT (Figure 5; Supplementary Figure S10). The majority of mutants differed strongly from wild-type, with abnormal progression of predicted CT and large, systematic errors. In five of seven datasets, absolute error was significantly higher for mutant than wild-type (unadjusted *P* < 0.05 by one-sided *t*-test; Figure 6A). The apparent severity and type of the defect in clock timing varied considerably from one mutant to another. For example, the liver clock of *Cry1^-/-^ Cry2^-/-^* mice was almost completely stuck near CT12, whereas the liver clock of liver-specific *Nr1d1^-/-^ Nr1d2^-/-^* mice appeared to progress normally. Of the three datasets with tissue-specific ablation of *Arntl*, timing defects in skeletal muscle and cardiomyocyte were similarly strong, whereas the timing defect in fat tissue was more subtle.

We hypothesized that a dysfunctional clock might cause not only aberrant timing, but also a poorer fit of the observed gene expression to what would be expected at a particular time. Consistent with this hypothesis, in four of seven datasets, log-likelihood of predicted CT was significantly lower in mutant than in wild-type (unadjusted *P* < 0.05 by one-sided *t*-test; Figure 6B). In particular, liver-specific *Nr1d1^-/-^ Nr1d2^-/-^* and fat-specific *Arntl^-/-^*, the two mutants that did not differ from wild-type in timing, differed unambiguously in log-likelihood.

Finally, we compared the location of wild-type and mutant samples in SPC-space to that of the training samples from GSE54650 (Figure 5B; Supplementary Figure S10). Wild-type samples in each dataset followed a similar trajectory to that of the training samples. In contrast, different mutants deviated from the trajectory of GSE54650 in different ways. For example, all *Cry1^-/-^ Cry2^-/-^* samples showed high expression of SPC1 and intermediate expression of SPC2. The circadian cycle in liver *Nr1d1^-/-^ Nr1d2^-/-^* was smaller than wild-type, whereas the circadian cycle in muscle *Arntl^-/-^* appeared to be shifted relative to wild-type. Thus, examining the samples in SPC-space reveals the basis for the differences in predicted CT and log-likelihood between wild-type and various mutants.

Because several of the genes knocked out in these datasets were used by the multi-organ predictor, it remained possible that our results were caused by lack of expression of the knocked out gene and not by the mutation's effect on the clock. To exclude this possibility, we repeated our analysis after removing the knocked out gene(s) in each respective dataset (Supplementary Figure S11). The results were very similar to those obtained without removing the genes. We conclude that the multi-organ predictor can sensitively detect when the circadian clock is dysfunctional.

## DISCUSSION

Supervised learning is a fundamental task in machine learning. ZeitZeiger is a supervised learning method specifically designed to take advantage of the special nature of periodic variables. Two aspects of ZeitZeiger's design are critical to its ability to make accurate predictions: regularization and focusing on the variation associated with the periodic variable. Regularization prevents overfitting, while focusing on the periodic variable prevents ‘misfitting.’

We speculate that a third aspect of ZeitZeiger's design will become relevant when ZeitZeiger is applied to data from other oscillatory systems, which is that ZeitZeiger captures the periodic behavior of the system in an unbiased way. Instead of assuming the features follow a sinusoid, ZeitZeiger uses a periodic smoothing spline (19). Although a sinusoid is a reasonable approximation for most circadian gene expression, it is likely a poor approximation for other periodic signals.

Besides making accurate predictions, our results highlight several other capabilities of ZeitZeiger. First, ZeitZeiger reveals the major oscillatory patterns in the data. When applied to GSE54650, ZeitZeiger automatically detected that the majority of circadian gene expression can be described by two patterns, consistent with earlier findings (25).

Second, ZeitZeiger identifies a small set of important features. The 13 genes of the multi-organ predictor, selected from 21 115 genes measured in GSE54650, include eight that are known to be part of the core circadian clock and two more that are known to be directly regulated by the clock. The three genes in the multi-organ predictor more loosely associated with the clock are *Fmo2, Lonrf3* and *Tsc22d3* (*Gilz*). *Fmo2* encodes a flavin-containing monooxygenase, but in most humans, the gene contains a premature stop codon and the enzyme is inactive (49). *Lonrf3* contains a Lon peptidase domain and a RING finger domain, but its function remains unclear. *Tsc22d3* is a glucocorticoid-induced leucine zipper protein that inhibits multiple signaling pathways and exerts a variety of effects on the immune system (50–52).

Third, the coefficients assigned by ZeitZeiger to a selected feature are suggestive of that feature's role in the oscillator. In the multi-organ predictor, the coefficients of the genes for the SPCs are consistent with their known functions in the circadian clock (27). For example, ARNTL and NPAS2, whose genes have negative coefficients for SPC1, form heterodimers that drive transcription of the genes that have a positive coefficient for SPC1.

Finally, ZeitZeiger can detect when the oscillator is phase-shifted or dysfunctional. If the oscillator is phase-shifted but otherwise functioning normally, observations will tend to have constant, non-zero prediction error and high log-likelihood. If the oscillator is dysfunctional (or functioning very differently than in the training data), observations will tend to have varying, non-zero prediction error and/or low log-likelihood.

This leads to a notable caveat of predicting a periodic variable, regardless of the method: the predicted value alone cannot indicate when the method is extrapolating. Because the set of possible values of the periodic variable is bounded, the predicted value for a test observation will always lie in the same range seen in the training observations. With ZeitZeiger, one can assess how close a test observation lies to the expected trajectory using the log-likelihood. If the log-likelihood is low (indicating extrapolation), one should check the observation's location in SPC-space relative to the trajectory of the training data.

In conclusion, we developed ZeitZeiger to enable regularized supervised learning on high-dimensional data from an oscillatory system. By applying ZeitZeiger to genome-wide gene expression related to the circadian oscillator, we created a multi-organ predictor of circadian time. Our results suggest that even with relatively small datasets, ZeitZeiger can make accurate predictions, identify major patterns and important features, and detect when the oscillator is perturbed. As our ability to collect high-dimensional data from various biological oscillators increases, we anticipate that ZeitZeiger will enhance efforts to understand and interrogate this important class of biological systems.

## AVAILABILITY

ZeitZeiger is available as an R package at https://github.com/jakejh/zeitzeiger. All code, data and results for this study are available at http://dx.doi.org/10.5061/dryad.hn8gp.

## Supplementary Material

SUPPLEMENTARY DATA
