# Supplementary material for: ZeitZeiger: supervised learning for high-dimensional data from an oscillatory system
Source: Nucleic Acids Res. 2016 Jan 26;44(8):e80. doi: 10.1093/nar/gkw030 (PMC4856978; doi:10.1093/nar/gkw030)
Supplement: SUPPLEMENTARY DATA [file supp_44_8_e80__index.html]

ZeitZeiger: supervised learning for high-dimensional data from an oscillatory system — ZeitZeiger: supervised learning for high-dimensional data from an oscillatory system — SUPPLEMENTARY DATA 

# ZeitZeiger: supervised learning for high-dimensional data from an oscillatory system

## SUPPLEMENTARY DATA

- SUPPLEMENTARY DATA
- SUPPLEMENTARY DATA
