# Supplementary material for: ZeitZeiger: supervised learning for high-dimensional data from an oscillatory system
Source: Nucleic Acids Res. 2016 Jan 26;44(8):e80. doi: 10.1093/nar/gkw030 (PMC4856978; doi:10.1093/nar/gkw030)
Supplement: SUPPLEMENTARY DATA [file supp_gkw030_nar-02950-met-n-2015-File012.pdf]

Supplementary Material for: ZeitZeiger: Supervised learning for high-dimensional data from an oscillatory system

Jacob J. Hughey[*,1], Trevor Hastie[2], and Atul J. Butte[1]

[1]Institute for Computational Health Sciences, University of California, San Francisco, San Francisco, CA 94158
[2]Department of Statistics, Stanford University, Stanford, CA 94305

[*]To whom correspondence should be addressed. Tel: +1 415 514 0511; Fax: +1 650 618 8605; Email: jakejhughey@gmail.com.

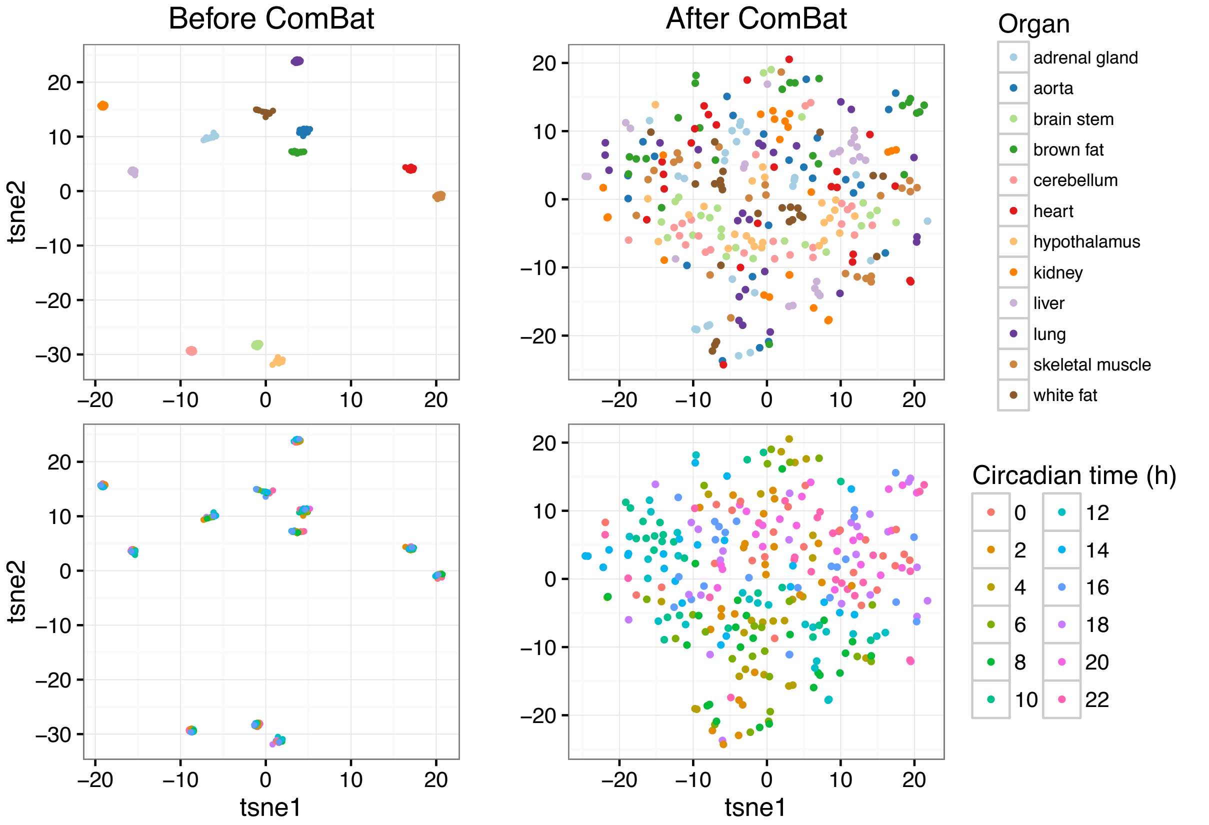

Supplementary Figure S1: ComBat corrects for the majority of organ-specific expression in samples from GSE54650. T-sne plots of gene expression before (left) and after (right) running ComBat. Each point is a sample from GSE54650, colored by organ (top) or circadian time (bottom). Before applying ComBat, the samples cluster strongly by organ. After applying ComBat, a subtle grouping of samples by circadian time is apparent (bottom right).

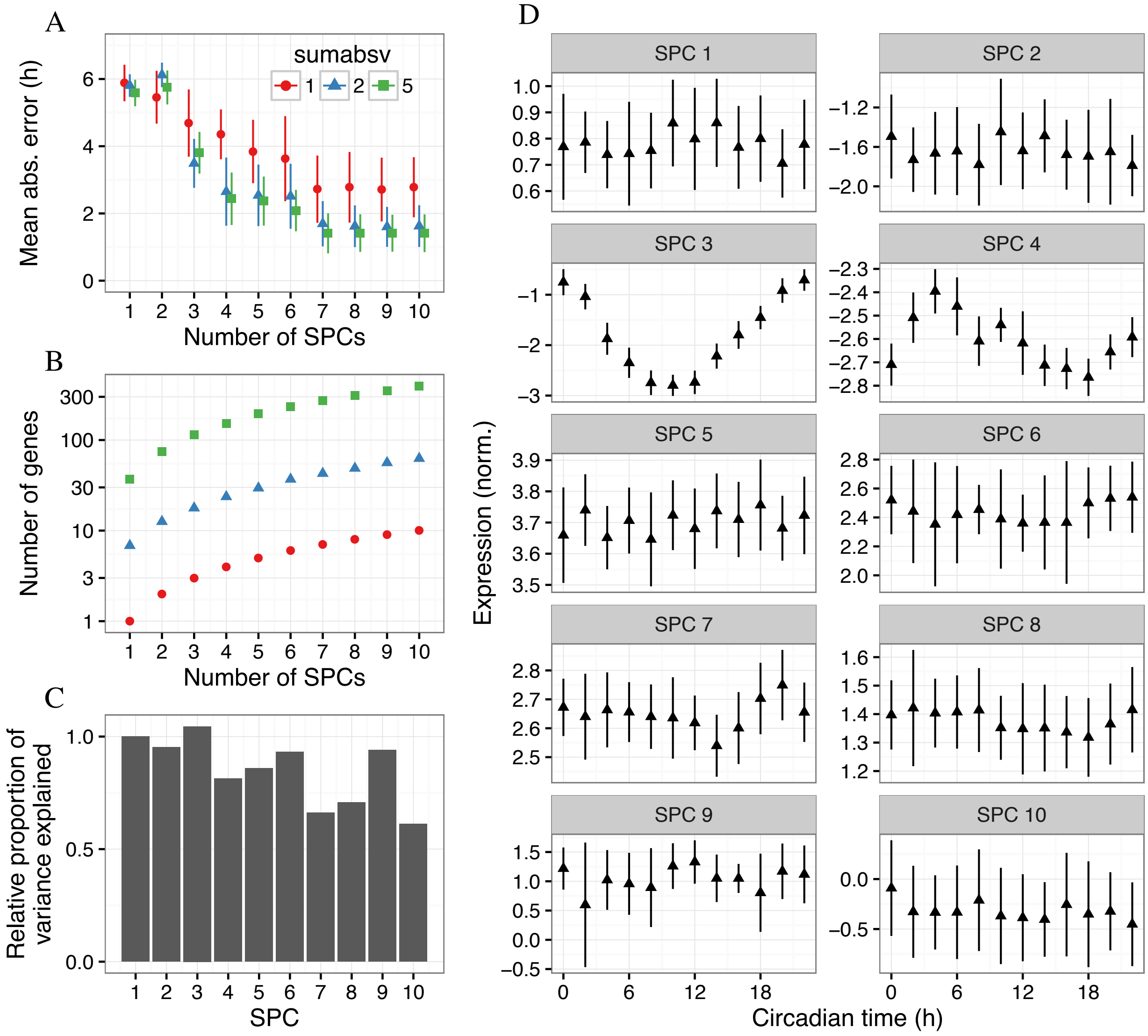

Supplementary Figure S2: Leave-one-organ-out cross-validation on samples from GSE54650, calculating the SPCs directly from the training data. As in the original cross-validation by ZeitZeiger, organ-specific differences in gene expression were adjusted using ComBat. In each fold of cross-validation, the expression of each gene was centered and scaled prior to calculating the SPCs. In each fold, the training data consists of 264 samples (288 - 24), but only the first 10 SPCs were calculated. (**A**) Mean absolute error vs. $nSPC$, for different values of $sumabsv$. For each parameter set, the point shows the overall mean absolute error across all 288 samples and the error bar shows the standard deviation of the mean absolute error across the 12 organs. The third, fourth, and seventh SPCs seem to improve prediction of circadian time, but not to the accuracy of ZeitZeiger. (**B**) Mean number of genes on cross-validation as a function of $sumabsv$ and $nSPC$. (**C**) Relative proportion of variance explained for the first 10 SPCs calculated using all samples from GSE54650 ($sumabsv = 2$). The SPCs are calculated using an iterative procedure, so the singular values are not constrained to be monotonically decreasing. (**D**) Expression of the first 10 SPCs ($sumabsv = 2$) vs. circadian time. The point corresponds to the mean and the error bar corresponds to the standard deviation. The third and fourth SPCs here are similar to the first and second SPCs calculated by ZeitZeiger, respectively, but the first and second SPC are not associated with circadian time at all.

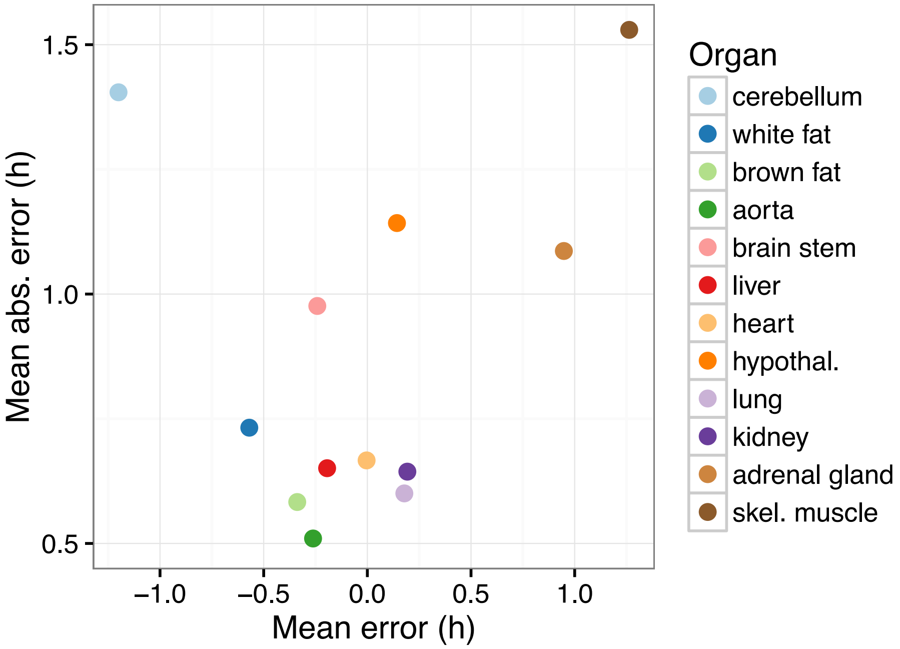

Supplementary Figure S3: Mean absolute error vs. mean error for each organ on cross-validation, based on training a ZeitZeiger predictor using $sumabsv = 2$ and $nSPC = 2$.

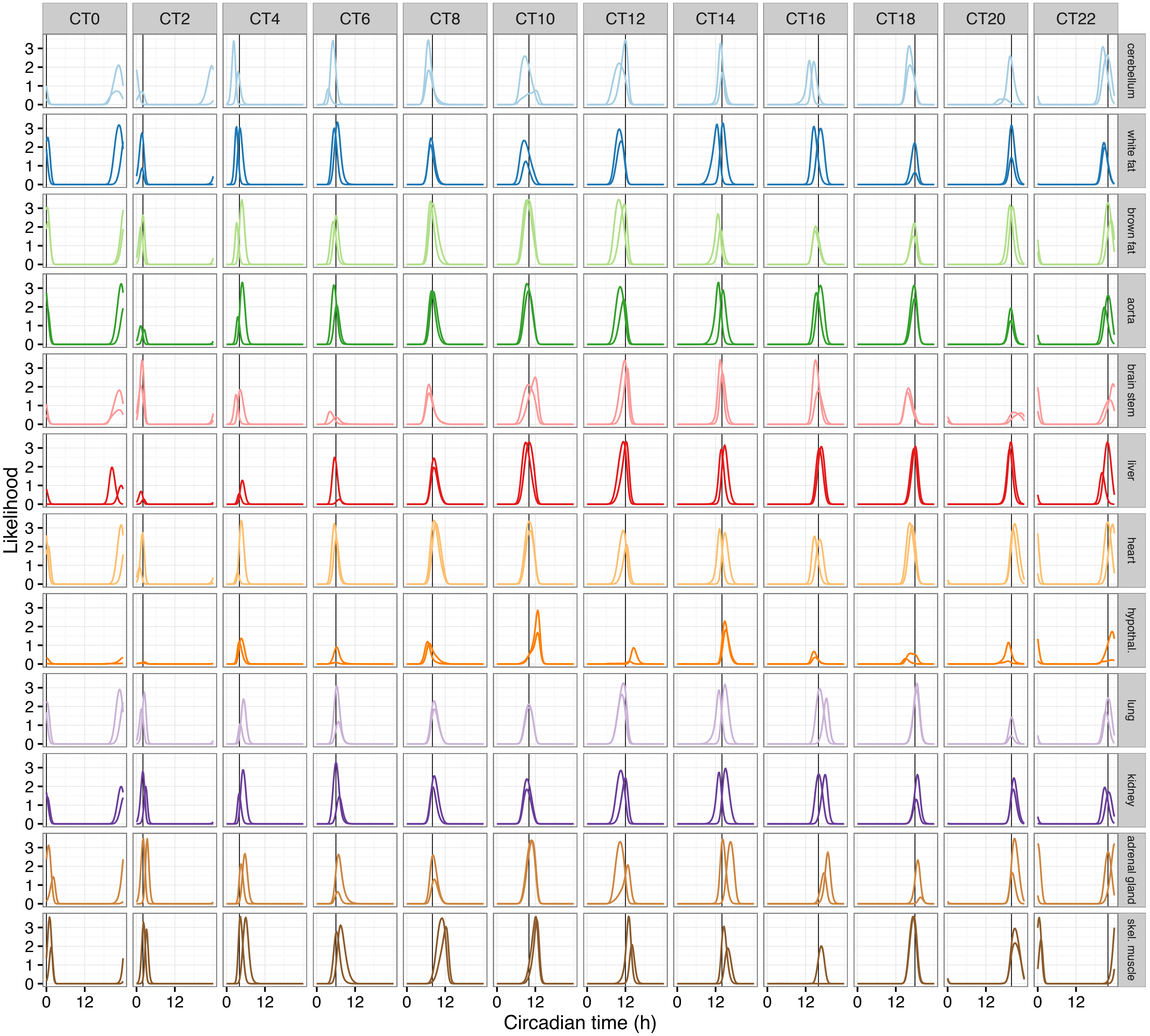

Supplementary Figure S4: Likelihood vs. circadian time for each sample of GSE54650 on leave-one-organ-out cross-validation ($sumabsv = 2$ and $nSPC = 2$). Each curve corresponds to one sample. The color of the curve corresponds to the organ. The vertical line in each plot corresponds to the observed circadian time for those samples. Each row shows samples from the same organ and each column shows samples from the same circadian time. The color and order of the organs are the same as in Figure 2C.

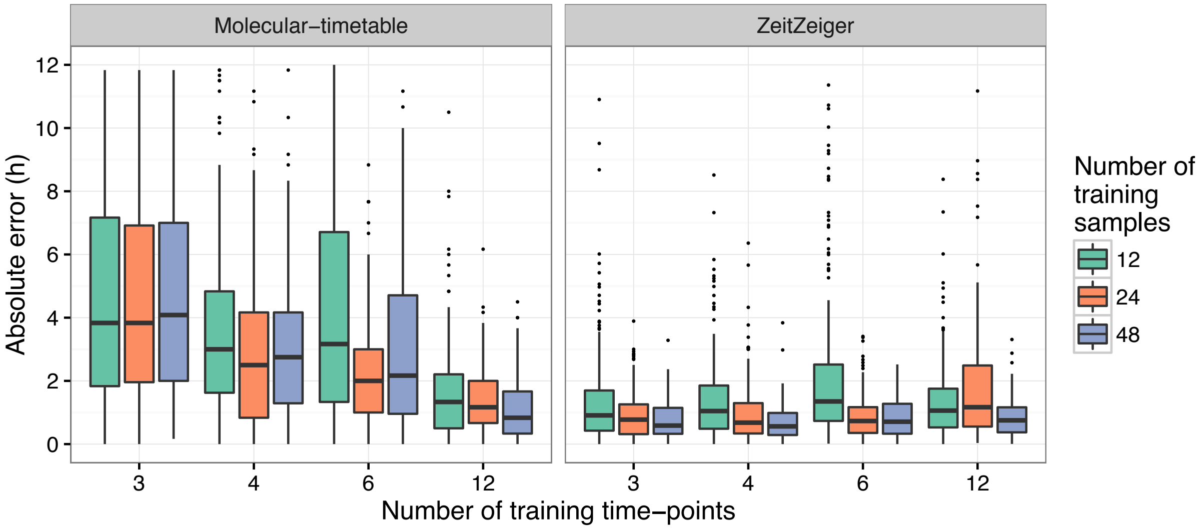

Supplementary Figure S5: Prediction accuracy for various numbers of samples and time-points in the training set. All ZeitZeiger predictors were trained using $sumabsv = 2$, $nSPC = 2$, and 4 knots for spline fitting (default is 30). All molecular-timetable predictors were trained using the same criteria for periodicity and variability that were used in Figure 2E. All training and test samples were from GSE54650. For analyses with 48 training samples, 8 organs from GSE54650 were randomly selected for the training set. Then, for analyses with 3 time-points, all 48 samples from CT0, CT8, and CT16 from the 8 training organs were used to train a predictor. For analyses with 4 time-points, 48 samples (out of a possible 64) were chosen from CT0, CT6, CT12, and CT18 from the 8 training organs. For 6 time-points, 48 samples were chosen from CT0, CT4, CT8, CT12, CT16, and CT20. For 12 time-points, 48 samples were chosen from CT0, CT2, CT4, etc. For all the above training sets, the test set consisted of all samples from the 4 organs not used in training (96 samples total). Analyses with 12 and 24 training samples were performed similarly, except that instead of selecting training samples from 8 organs, training samples were selected from 2 and 4 organs, respectively.

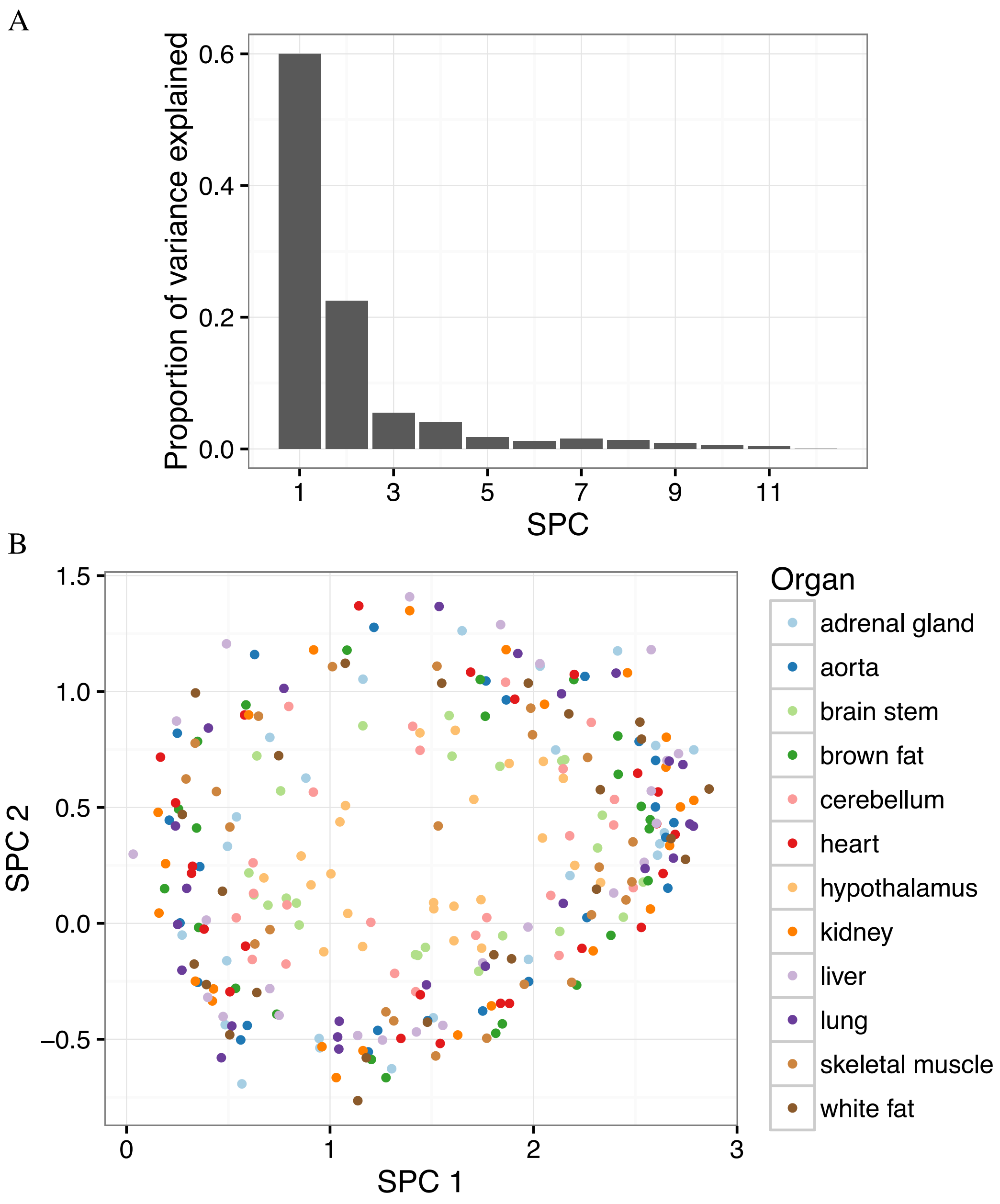

Supplementary Figure S6: Properties of the sparse principal components (SPCs) calculated by ZeitZeiger ($sumabsv = 2$) based on samples from GSE54650. (**A**) Proportion of variance explained for each of the 12 SPCs. The first two SPCs explain over 80% of the variance. (**B**) Gene expression of samples in SPC-space, colored by organ. Compare to Figure 2B. Samples from brain stem, cerebellum, and hypothalamus are mostly on the inside of the limit cycle, indicating that circadian oscillations (as measured by gene expression) in those organs are weaker than in the other organs.

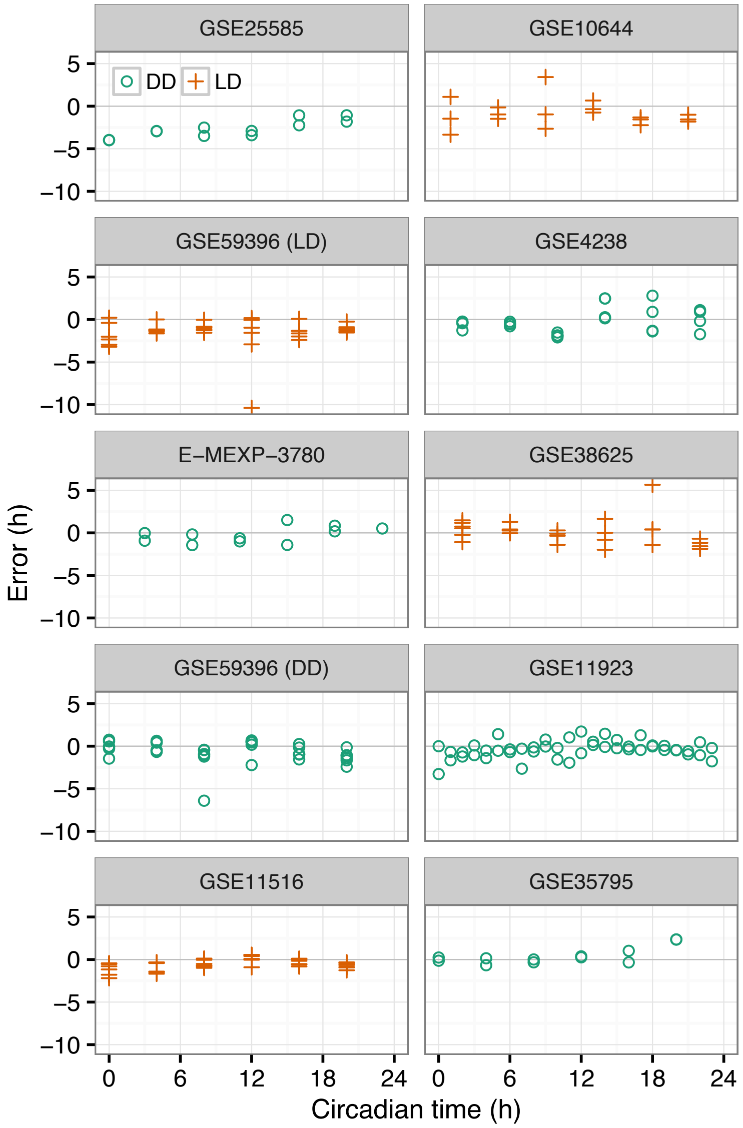

Supplementary Figure S7: Prediction error vs. circadian time for samples from datasets in which circadian gene expression was measured in wild-type mice. Each point is a sample, with the style of the point indicating the light:dark regimen for that sample. Datasets are sorted by median absolute error. See Table 1 or Supplementary Table S1 for more information on the datasets.

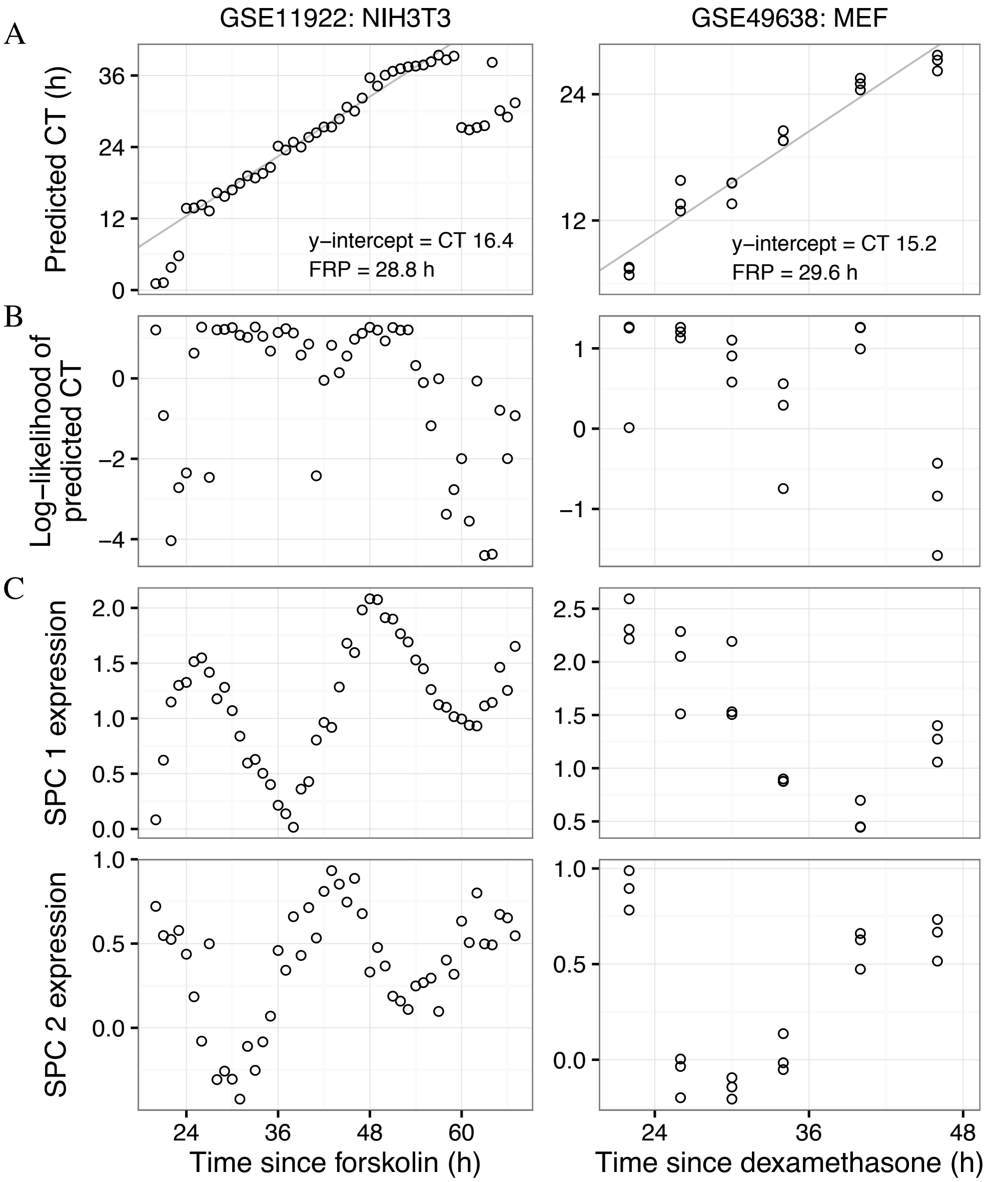

Supplementary Figure S8: Applying the multi-organ predictor to two datasets of gene expression from mouse fibroblasts cultured *in vitro*. Each point is a sample. (**A**) Predicted circadian time vs. time since synchronization. For ease of visualization, the predicted circadian time of the later time-points in both datasets is shifted by 24 hours. For GSE11922, the linear fit was based on samples with time since forskolin greater than or equal to 24 h and less than 60 h. For GSE49638, the linear fit was based on all samples. The correlations of the linear fits were $R = 0.99$ for GSE11922 and $R = 0.97$ for GSE49638. (**B**) Log-likelihood of predicted circadian time vs. time since synchronization. Greater log-likelihood indicates a better fit to the expected gene expression for that circadian time. The predicted circadian times of the first and last few samples for GSE11922 had a much lower log-likelihood, indicating that ZeitZeiger recognized that the gene expression of those samples did not correspond well to any circadian time. (**C**) Expression of SPC1 and SPC2 vs. time since synchronization. Based on the expression of the SPCs in each dataset, the drop in log-likelihood at later times may be a result of the circadian clock in individual cells becoming desynchronized.

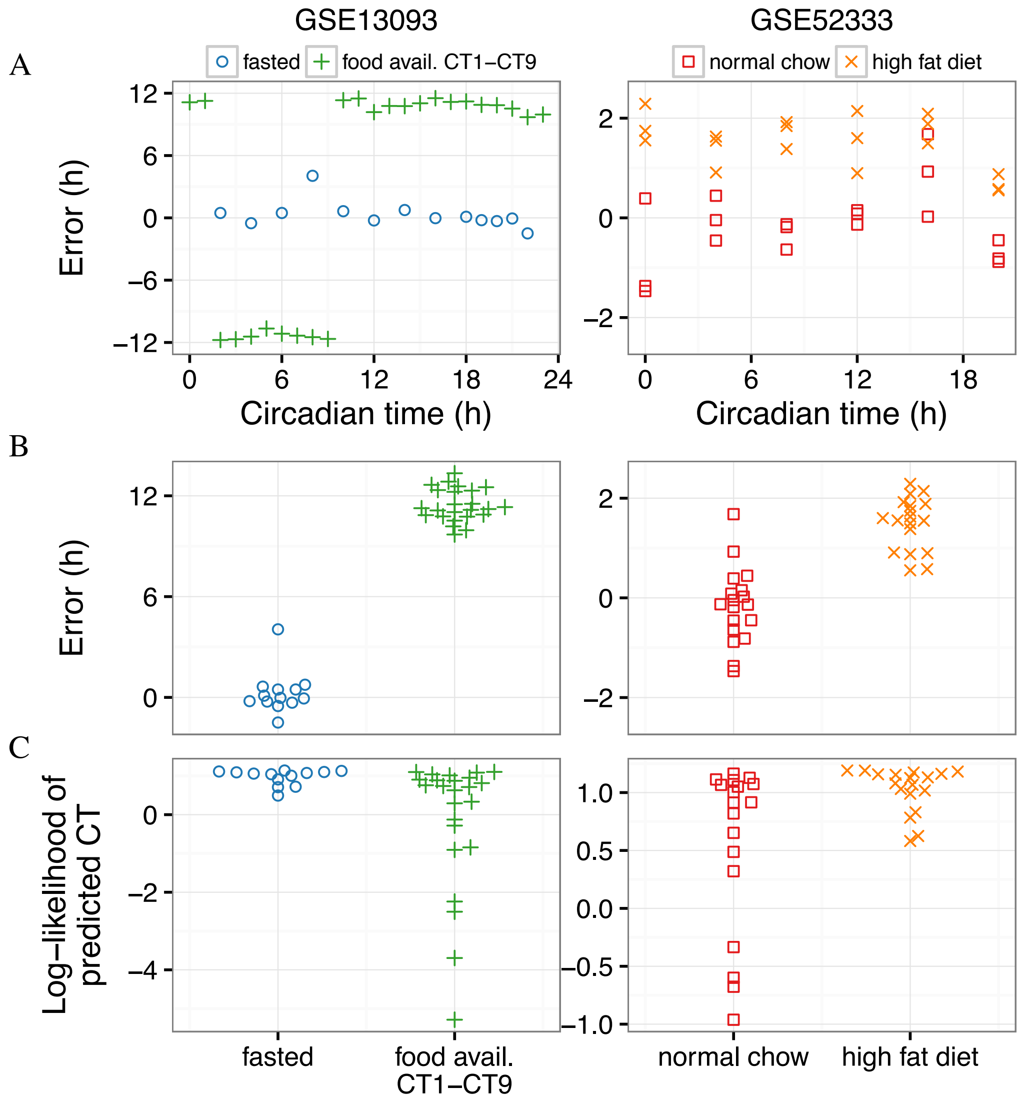

Supplementary Figure S9: Applying the multi-organ predictor to gene expression from livers of mice subject to dietary perturbations. Each point is a sample, with the style of the point indicating the dietary condition. (**A**) Prediction error vs. circadian time for samples from two datasets. (**B**) Distributions of error for the two conditions in each dataset. For GSE13093, errors less than zero were shifted by 24 hours. (**C**) Distributions of log-likelihood of predicted circadian time for the two conditions in each dataset.

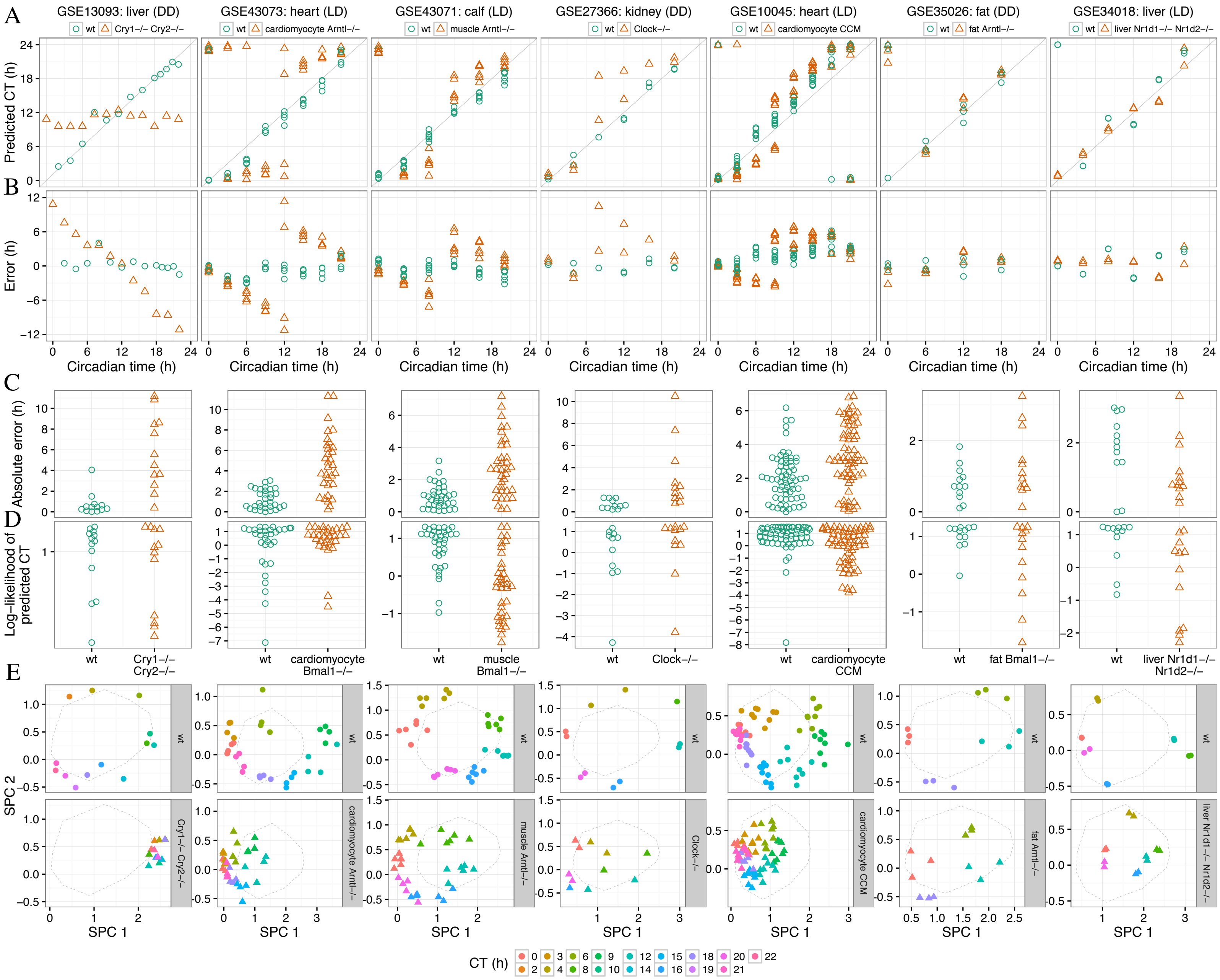

Supplementary Figure S10: Analysis of gene expression from mice with a genetically perturbed circadian clock. Each column shows the results from one dataset. Each point is a sample. In **A-D**, the style of the point corresponds to the sample's genotype (wild-type or mutant). In (**E**), the color of the point corresponds to the sample's circadian time. (**A**) Predicted circadian time vs. circadian time. (**B**) Prediction error vs. circadian time. (**C**) Distribution of absolute error for wild-type and mutant genotypes. (**D**) Distribution of log-likelihood of predicted circadian time for wild-type and mutant genotypes. (**E**) Gene expression of wild-type (upper) and mutant (lower) samples in SPC-space. The dashed line shows the mean trajectory of the training samples from GSE54650. The mean trajectory of the training samples is slightly different for each dataset, because each dataset is merged with GSE54650 separately, so the cross-study normalization is slightly different for each dataset.

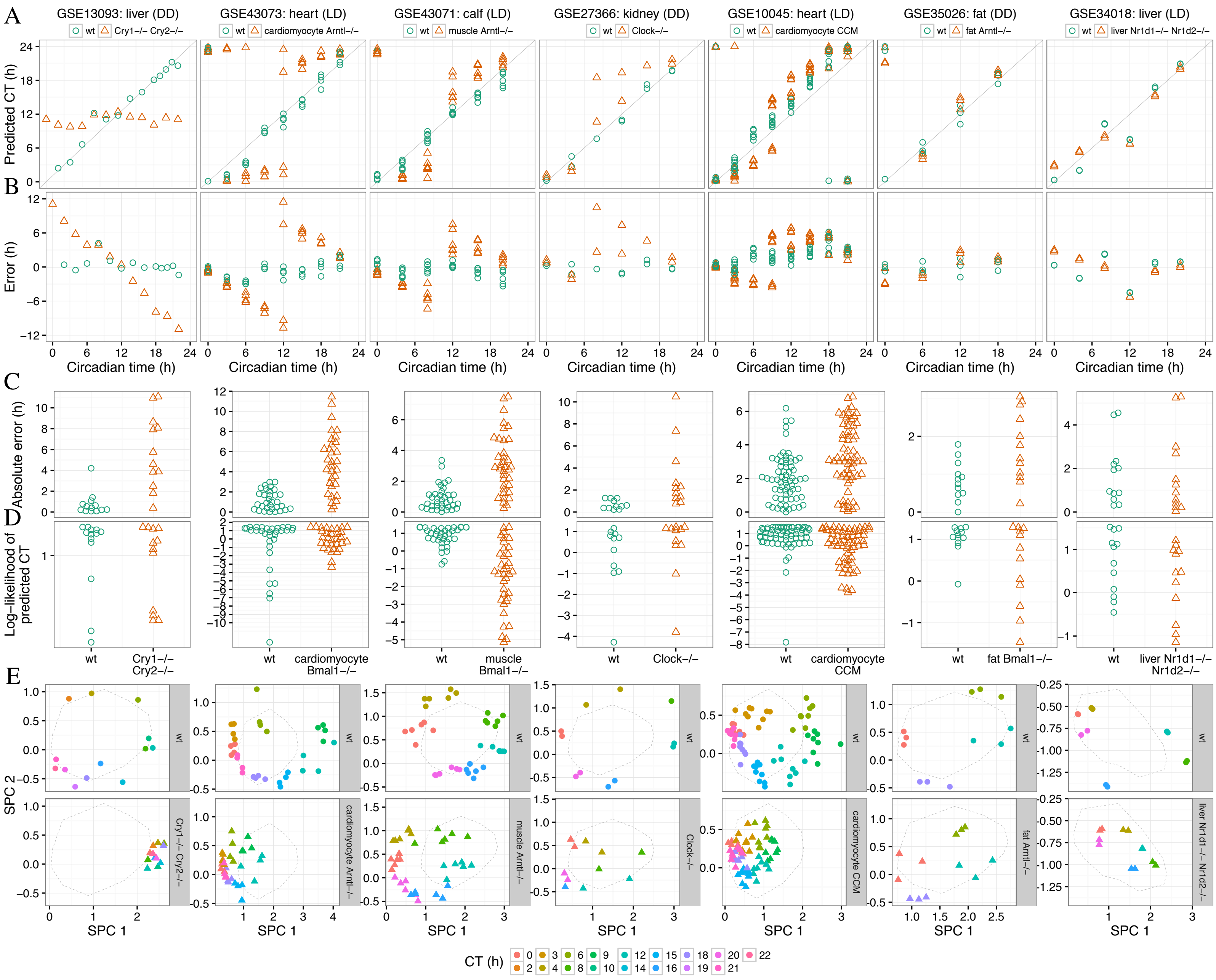

Supplementary Figure S11: Analysis of gene expression from mice with a genetically perturbed circadian clock, here excluding the knocked out (or otherwise perturbed, in the case of GSE10045) gene(s). For example, when analyzing GSE13093, both *Cry1* and *Cry2* were removed from the expression profiles of the training samples in GSE54650, and the wild-type and mutant samples in GSE13093. Compare to Supplementary Figure S9. The results for each dataset are almost identical to those obtained without removing the genes, with the exception of GSE34018, where the log-likelihood of predicted time for wild-type samples has dropped.
